# Classification of extremity movements by visual observation of signals and their transforms

**DOI:** 10.1016/j.mex.2022.101739

**Published:** 2022-05-27

**Authors:** Manuel Enrique Hernandez, Liran Ziegelman, Tanvi Kosuri, Husain Hakim, Luqi Zhao, Kelly Alexander Mills, James Robert Brašić

**Affiliations:** a Department of Kinesiology and Community Health, University of Illinois at Urbana-Champaign, Urbana, IL 61801, United States; b Department of Public Health Studies, Krieger School of Arts and Sciences, The Johns Hopkins University, Baltimore, MD 21218, United States; c Department of Neuroscience, Krieger School of Arts and Sciences, The Johns Hopkins University, Baltimore, MD 21218, United States; d Department of Neurology, The Johns Hopkins University School of Medicine, Baltimore, MD 21205, United States; e Section of High Resolution Brain Positron Emission Tomography Imaging, Division of Nuclear Medicine and Molecular Imaging, The Russell H. Morgan Department of Radiology and Radiological Science, The Johns Hopkins University School of Medicine, Baltimore, MD 21205, United States

**Keywords:** Accelerometer, Continuous wavelet analysis, Experimental error, Fourier analysis, Inertia measurement units, Movement disorders, Multiple system atrophy, Rating scales, Typical development, Wearable sensors

## Abstract

A low-cost quantitative continuous measurement of movements utilizes accelerometers to generate signal outputs to precisely record the positions of extremities during the performance of movements. This procedure can readily be accomplished with inexpensive materials constructed indivisuals throughout the world. The proposed protocol provides the framework for trained raters to assess the signal outputs by visual observation to generate objective measurements like the measurements of the actual movements. Expert raters can then remotely give quantitative suggestions for providers in underserved regions to utilize precision medicine to develop optimal treatment plans tailored to the specific needs of each individual. The proposed protocol lays the foundations for experts located in tertiary centers to provide optimal assessments of signal outputs generated remotely in underserved regions. This protocol provides the means to address gaps in current research including the dearth of objective measurements of movements utilizing automatic intelligence and machine learning to accurately and precisely analyze movement assessments. Future research will include the development of robotic tools to perform assessments and analyses of the movements of human beings to enhance the conduct of movement evaluations of people with Parkinson's disease and related conditions to apply precision medicine for optimal diagnostic and therapeutic interventions.

## Graphical abstract

**Figure:**
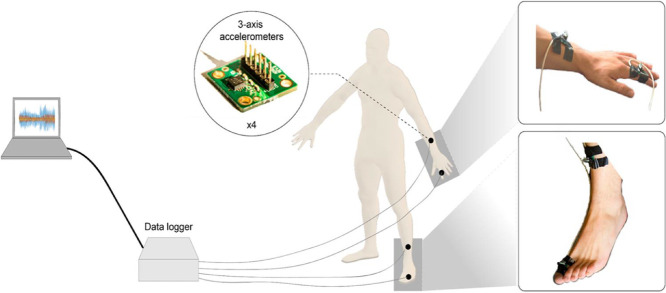

Specifications table

**Table:** 

**Subject Area**	Medicine and Dentistry
**More specific subject area**	Neurology
**Protocol name**	A low-cost quantitative continuous measurement of movements in the extremities of people with Parkinson's disease
**Reagents/tools**	MATLAB [software] (2022). Natick, MA: Mathworks. https://www.mathworks.com/products/matlab.html.
**Experimental design**	Signals generated by accelerometers on the extremities of people with Parkinson's disease and the transforms of the signals were represented as images to be assessed by trained raters using scoring procedures analogous to live in-person assessments.
**Trial registration**	Not applicable
**Ethics**	Written informed consent was obtained from all participants utilizing a protocol (IRB00110166) approved by Johns Hopkins Medical Institutions Institutional Review Board, Baltimore, Maryland, USA.
**Value of the Protocol**	• Providers in remote and underserved regions can readily generate signals to facilitate the diagnosis and treatment of Parkinson's disease and other movement disorders. • Output of a low-cost quantitative continuous measurement of movements can be interpreted by trained raters remotely. • Experts who review the signals and their transforms from this protocol can guide providers around the world to utilize precision medicine to generate optimal treatment plans for people with Parkinson's disease and related conditions.

## Method details

### Rationale

Parkinson's disease (PD) is manifested clinically by tremor, rigidity, bradykinesia, and postural instability and pathologically by alpha-synuclein-containing Lewy bodies and reduced dopaminergic neurons in the substantia nigra. The incidence of PD, a condition afflicting 1% of people aged 60 years and older throughout the world, increases with age [1]. The evaluation of individuals with possible PD includes a comprehensive assessment of the medical and family histories, the physical and neurological examinations, and laboratory evaluations [2,3]. The administration of structured assessments to provide quantitative measurements constitutes a key component for the diagnosis and treatment of people with PD and related conditions. The Movement Disorders Society-sponsored revision of the Unified Parkinson's Disease Rating Scale (MDS-UPDRS) [4], the gold-standard tool for clinical and research purposes, requires the examiner to differentiate minute distances in the movements of the patient by visual observation while the examiner is positioned several feet from the patient. Therefore, experimental error in the assessments is inherent in this tool utilizing human perception of movements at a distance from the participants. For this reason, we developed tools to employ instrumentation capable of detecting and recording fine variations in human movements to generate accurate and precise measurements for standard ratings. Thus, we developed a low-cost quantitative continuous measurement of movements in the extremities of people with Parkinson's disease [5] to provide the means to express the dysfunction of movements commonly seen in people with PD in the form of the signals of accelerometers capturing the three-dimensional position in space of the extremities during movements. Our low-cost quantitative continuous measurement of movements in the extremities of people with Parkinson's disease provides the means to assess the dysfunction of participants by ratings of the movements using visual observation of the live behaviors (in person and video) and the output of the instrumentation [5], [6], [7]. The goal of the current protocol is to provide the means for trained raters to remotely provide objective assessments of the signals and their transforms of the output of our low-cost quantitative continuous measurement of movements in the extremities of people with Parkinson's disease without observing the participant performance of the tasks [5], [6], [7]. To attain this end, we sought to develop a method for 35 experts certified in the MDS-UPDRS [4] to blindly rate the signals and transforms of our quantitative continuous measurement of movements in the extremities of cohorts of people with PD and control and comparison groups [5], [6], [7], [8], [9], [10], [11].

Thus, we applied our accelerometry-based method for the acquisition of motion data for 12 tasks that demonstrate signs of PD [5] on 20 patients with PD, one patient with multiple system atrophy (MSA), a condition with some traits characteristic of PD, and eight healthy age- and sex-matched healthy individuals with typical development (TD) [11]. The original output from the instrumentation was stored on the laptop used for the study. The original hand-written scores of the full live in-person person ratings as well as the signals and their fast Fourier transforms (FFTs) and continuous wavelet transforms (CWTs) have been published [6,7]. The videos of the live assessments are being prepared for publication. However, the published materials express data in varying formats that cannot be correlated for blind ratings by experts without access to the original data because the published data are in separate datasets [6], [7], [8], [9] that cannot easily be combined by humans using visual observation. For this reason, we sought to develop a protocol to express the signals and the transforms of the five repetitive movements (3.4 Finger tapping, 3.5 Hand movements, 3.6 Pronation-supination movements of hands, 3.7 Toe tapping, and 3.8 Leg agility) [4,5] of each of the cohorts in a format suitable for blind rating by experts unfamiliar with the original data [11], [12], [13].

The work was carried out in accordance with the recommendations of the World Medical Association in the WMA Declaration of Helsinki - Ethical Principles for Medical Research Involving Human Subjects [20] and the Recommendations for the Conduct, Reporting, Editing, and Publication of Scholarly Work in Medical Journals [21]. The study was approved by the Institutional Review Board of The Johns Hopkins University School of Medicine in Baltimore, Maryland, United States. All participants provided written informed consent to take part in this study. Portions of the MDS-UPDRS [4] were utilized with the kind permission of the International Parkinson and Movement Disorder Society.

### Procedure

#### Raters

Due to the worldwide health crisis meetings of raters in person were forbidden during the conduct of the investigation. Therefore, experts from all around the world were invited to take part in the investigation as trained raters of the signals and their transforms already generated by the cohorts in the original study[[5], [6]]. To verify that each rater was qualified to participate as a rater, each potential rater was required to join the International Parkinson and Movement Disorder Society to obtain certification in the MDS-UPDRS [4]. Additionally, potential raters participated in weekly online research team meetings including instruction by a biomedical engineer and a mechanical engineer about the expression of output signals as FFTs and CWTs. Throughout the team meetings conducted during the rating months, the criteria to score signals and transforms analogous to those for live ratings were repeatedly expressed. However, raters were instructed to ultimately use their best judgment to apply the indicated criteria to the presented images even though the images may not optimally match the indicated scales for scores. Raters were asked to perform ratings independently without consultation with others. Because the research team of expert raters included people throughout the world, raters were allowed to view the images as long as they wanted. They were allowed to change ratings. They could take as much time as they wanted to complete and submit each set of ratings. The full set of ratings was completed by 35 trained raters certified in the MDS-UPDRS [4,[6], [7], [8], [9], [10], [11]].

#### Rating protocol

To develop a procedure to rate by visual observation the signals of movements and their transforms with a procedure analogous to the rating of the movements themselves directly by visual observation, we constructed images of the individual movements of each procedure in each extremity for each participant. Throughout all image representations, separate panels were constructed to express the signal and its FFT or the CWT without the signal for assessments by trained raters. The images were presented randomly without identification of laterality (left or right) or status (PD, MSA, or TD) for all rating sessions. For each rating session, raters were given the output representation (signal and its FFT [12]) or (CWT without its signal [13]), the location [(finger and wrist) or (ankle and toe)], and the task (3.4 Finger tapping, 3.5 Hand movements, 3.6 Pronation-supination movements of hands, 3.7 Toe tapping, 3.8 Leg agility) [4,^5^,18] of individual participants without identification of demographic characteristics, clinical status, or laterality.

**Fig. 1:**
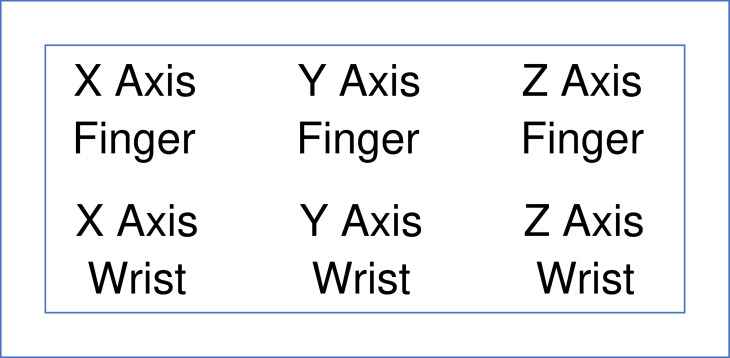
Schematic representation of the images for the x axis (left column), y axis (middle column), and z axis (right column) of the output for the finger (top row) and wrist (bottom row).

**Fig. 2:**
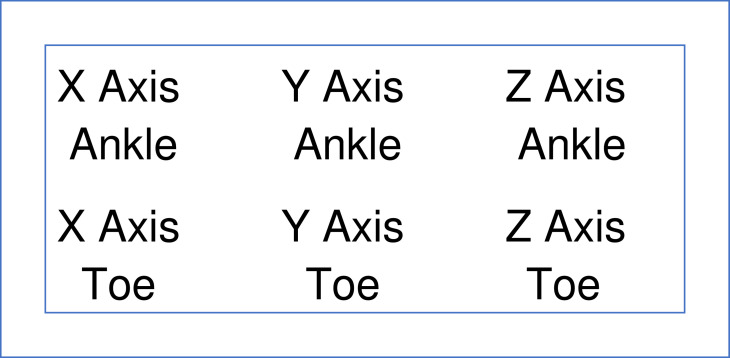
Schematic representation of the images for the x axis (left column), y axis (middle column), and z axis (right column) of the output for the ankle (top row) and toe (bottom row).

Specific protocols were constructed for the random presentations of test and retest sessions of the individual cohorts [single assessment of MSA, single assessment of PD, two assessments of PD or TD randomly presented without identification of status (PD or TD)]. The images of the person with MSA and the persons with PD with single assessments were presented randomly to raters as panels with six images of the x, y, and z positions of either the finger (upper row) and wrist (lower row) (Fig. 1) or the ankle (upper row) and toe (lower row) (Fig. 2) for separate ratings [9,11]. Below the images were spaces to record the presence or absence of the possible abnormalities observed in the images [11].

**Fig. 3:**
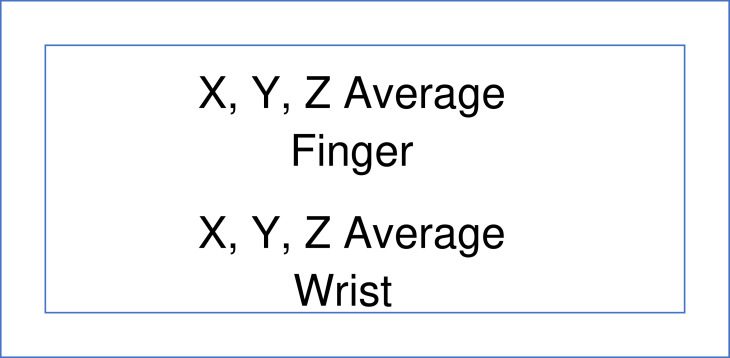
Schematic representation of the images for the average of the x, y, and z axes of the output for the finger (top row) and wrist (bottom row).

**Fig. 4:**
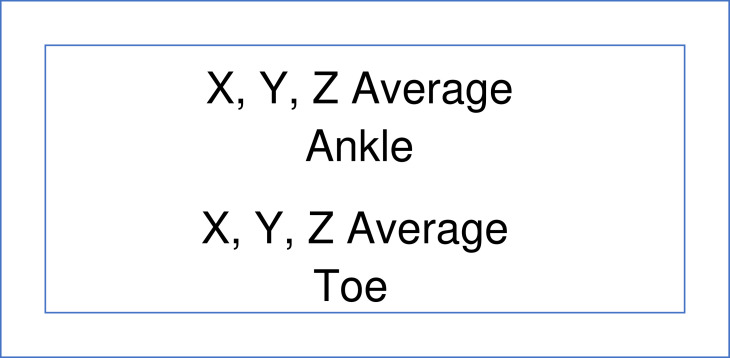
Schematic representation of the images for the average of the x, y, and z axes of the output for the ankle (top row) and toe (bottom row).

The images of the persons with PD or TD with two assessments were presented without identification of status (PD or TD) as two images representing averaged representations of the x, y, and z axes for the finger and wrist (Fig. 3) or the ankle and the toe (Fig. 4) in two sequential groups for raters to complete before proceeding to the next [11]. Below the images were spaces to record the presence or absence of the possible abnormalities observed in the images [11].

Raters were required to complete each rating session in sequence (MSA with single session, PD with single session, PD and TD without identification of status (PD or TD) in two sequential groups [9].

#### Rating instructions

Because the study was conducted during the pandemic when meetings in person were not allowed, the entire process was completed online by raters around the world. Raters were instructed to complete all ratings independently without consultation with anyone else. Since the raters were located around the world, they were allowed to complete the ratings at their convenience whenever they liked. They were allowed to take as much time to view images as they wanted. They were allowed to change rating scores. Once all ratings in a set were completed, the raters submitted the scores to the administrators. The scores could not then be changed. The specific instructions to rate the images [(signal traces and FFTs) or (CWTs without signal traces)] were modified from those for the live ratings of movements [4,5]. Raters were instructed to score images for no abnormalities as 0 (zero) and for abnormalities as follows [11], [12], [13]:

A. Interruptions or freezing

 1a 1 to 2 interruptions

 2a 3 to 5 interruptions

 3a 5 or more interruptions or a freeze, a sustained absence of repetitions

 4a  Worse

B. Slowing

 1b Minimal

 2b Mild

 3b Moderate

 4b Worse

C. Amplitude reductions

 1c End of sequence

 2c Middle of sequence

 3c Beginning of sequence

 4c Worse

## Method validation

The method was applied to the cohorts of participants with PD, MSA, and TD. Signal processing algorithms [14,16] were written to extract and analyze the data. FFTs [15] and CWTs [17] were applied to the output of the five repetitive movements of all participants. Single test analysis was applied to the signals of one man with MSA and ten participants with PD. Test sessions with a repeat test session a month or so later was performed on 20 participants with Parkinson's disease and eight healthy control participants with TD [9]. These data are being analyzed for future publications.

Raters were given representations as six images of the output of each extremity of the man with MSA as test and retest expressions [9,11]. They were randomly presented to all raters in four random orders, resulting in the evaluation of two signal and FFT representations and two CWT without signal representations [9]. Raters were required to complete the ratings of the man with MSA before proceeding to the next project.

Raters were then asked to complete ratings of representations as six images of the output of each extremity of ten participants with PD who completed single rating sessions [9,11].

Raters were required to complete the ratings of 40 FFT representations and 40 CWT representations from the ten participants with PD who completed single ratings before proceeding to the next project [9].

Raters were then asked to complete ratings of representations as two images of the output of each extremity of 20 participants with PD and eight age- and sex-matched healthy participants with TD who completed two rating sessions [9]. Overall, raters were required to complete the ratings of 148 CWT representations in a counterbalanced pseudorandomized design that controlled for the difficulty of the ratings by balancing the level of motor impairment across presentations [9].

## Limitations

### Participants

The participants were recruited from the clinical practices of collaborating neurologists who specialize in movement disorders. The diagnoses of the participants with PD and MSA were determined by the referring movement disorders neurologists [[5], [6], [7]]. The participants with TD were recruited from the cohorts of healthy controls with TD who had taken part in other investigations and from the colleagues and co-workers of the investigators. The records of each participant were supplemented by interviews for each testing session. Toward the end of the study measurements of the forearm were obtained to estimate the lever arm of movements. No additional interview, examination, or laboratory assessment was conducted for each live rating session.

### Treatment status

Although clinical records were obtained on each participant, the treatment status of each participant was recorded only midway during the testing process. After the study had started, raters began to ask and record the status (off, on) for levodopa/carbidopa medication and for deep brain stimulations (DBS).

### Rater training

Although each evaluation was conducted by an examiner who is certified in the MDS-UPDRS [4,5], interrater and test-retest reliability of the examiners were not performed. The scores of the live clinical assessments were determined by a single examiner certified in the MDS-UPDRS [[4], [5]].

### Rating procedure

All ratings were conducted with the participants in straight-back chairs with arms and without wheels. However, the ratings were conducted with different chairs in different rooms with variable ambient temperature. Although the twelve tasks of movements affected in PD were obtained for all ratings, the current protocol was limited by the use of only the five repetitive items in the low-cost quantitative continuous measurement of movements in the extremities of people with Parkinson's disease [4,^5^,18].

### Signal processing

The segments of the recorded signals utilized included sections before and after the start of the task. Therefore, the studied segments included beginning and ending sections that do not reflect the studied tasks. Additionally, the recorded segments included sixty or more repetitions of the study movement. The initial section of the repetitive task performed by the participant before the examiner repeated the command to perform the task as quickly and fully as possible were included in the study segments. Thus, the study segments include much more than the ten repetitions utilized in the MDS-UPDRS [4,5].

### Uniform rating sessions

The current investigation to rate signals and their transforms was limited by the lack of uniformity of the rating procedure by raters. Since the raters were located around the world, they were allowed to perform the ratings at their convenience. They were allowed to take as much time as they liked. They were allowed to review ratings to change them before submitting them for scoring.

## Future directions

Future investigations will study the signals and transforms of all twelve tasks of the low-cost quantitative continuous measurement of movements in the extremities of people with Parkinson's disease [5]. Future investigations will correlate the ratings of the signals and transforms with the clinical ratings performed by the examiners at the time of the live assessment and with the videos of the clinical ratings.

Future investigations will be enhanced by conducting interview, examination, and laboratory assessment of each participant at the time of each rating session. At each future assessment the examiner will determine and record the specific clinical status of each participant, including concurrent medications and the on or off status of levodopa/carbidopa medication and deep brain stimulation (DBS). In addition to the height and weight on the day of each assessment, measurements will be made of the length of the forearm to estimate the lever arm for movements. Future ratings will be conducted with a single straight-back chair with arms and without wheels in a set room with a specific temperature. All ratings will be conducted by a single examiner to provide uniformity of administration. All ratings will be recorded on video for subsequent blind rating by trained raters to evaluate interrater reliability. All twelve tasks of the low-cost quantitative continuous measurement of movements will be performed, recorded, and analyzed for future investigations.

In future studies raters will be required at attain certification in the MDS-UPDRS annually to maintain eligibility to perform assessments as trained raters. The videos obtained will be blindly and independently scored by trained raters certified in the MDS-UPDRS to assess the validity of the original live ratings.

Future investigations may be enhanced by requiring all raters to perform the ratings in person without communication with other raters immediately after a single presentation of the study images. Raters will be positioned so that they all have similar views of the images for set periods of observation. This will provide the foundation for the assumption that the rating values were independently and identically distributed.

*Machine learning to identify pathognomonic characteristics of signals and transforms*

Automatic intelligence (AI) will be applied to the machine learning of the analysis of the CWT images to generate a tool for hardware to provide a quantitative assessment of the output without the errors of human visual observation [22,23].

### Portable sensors

The current study was limited by use of accelerometers connected by wires to the participant. This arrangement limits the use of the procedure for stationary assessments in the clinic. The use of wearable accelerometers [24] will facilitate the development of this procedure for remotely. Future investigations will include inertial sensors[19], compasses, electromyograms (EMGs), gyroscopes, systems combining inertial sensor techniques, force sensors, gait analysis, and ground platforms.

### Clinical applications

**Fig. 5:**
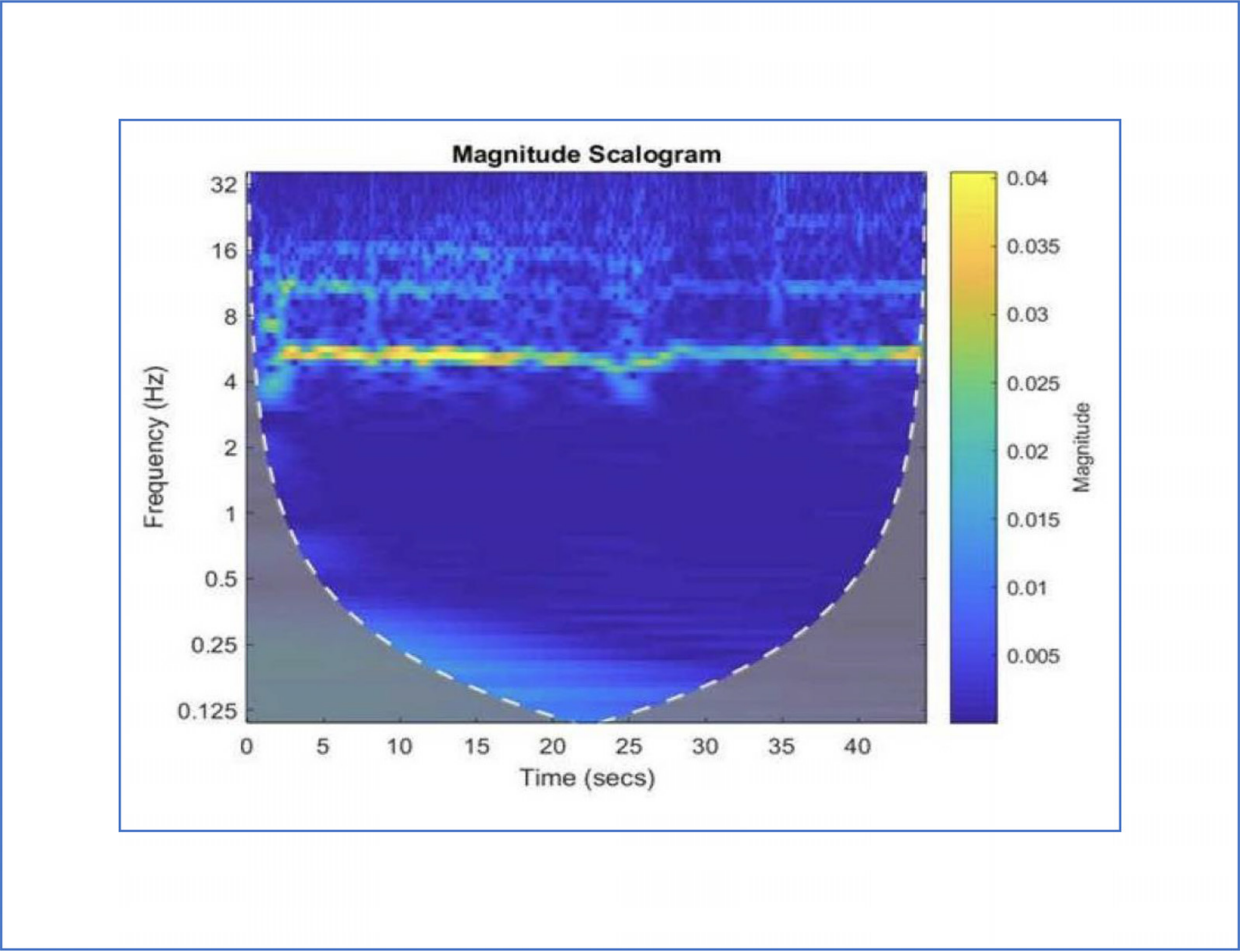
Continuous wavelet transform (CWT) of a mild tremor simulated by a healthy 25-year-old man with typical development (TD). The baseline frequency (6 Hz) and harmonics at 12 Hz and 18 Hz are regularly demonstrated. The y axis provides measurements of the frequencies. The x axis provides measurement of time. The color of the images provides a measurement of magnitudes according to the scale on the right [25].

**Fig. 6:**
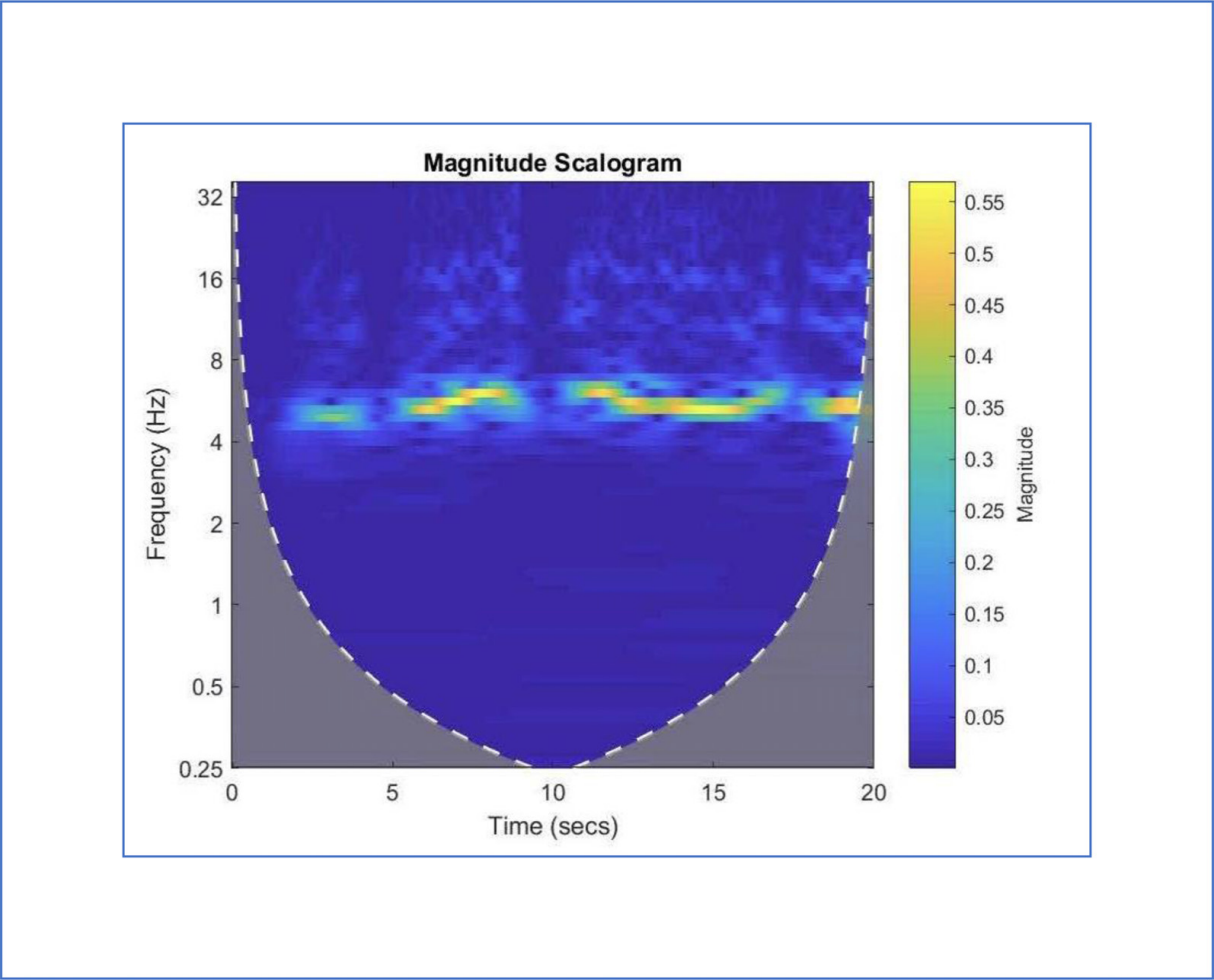
Continuous wavelet transform (CWT) of a severe tremor simulated by a healthy 25-year-old man with typical development (TD). The interruptions, freezes, slowing, and amplitude decrements are well demonstrated in this picture. The baseline frequency (6 Hz) and harmonics at 12 Hz and 18 Hz are interrupted at 5, 10 and 18 seconds. The y axis provides the frequencies. The x axis provides measurement of time. The color of the images provides a measurement of magnitudes according to the scale on the right [25].

Representations of the output from the instrumentation of this protocol can by utilized by clinicians to monitor the progress of patients with PD and related conditions. The images of the signals, their FFTs [12], and their CWTs [13] provide concise records of the status of patients. Therefore, these tools provide the means to obtain measurements of the key functions of patients with PD throughout their clinical course. The images provide concise means to assess participants before, during, and after clinical trials of interventions for PD. The potential usefulness of CWTs can be visualized in the images of mild (Fig. 5) and severe tremors (Fig. 6) simulated by a healthy 25-year-old man with typical development (TD). Frequencies are indicated by the y axis. Time is recorded on the x axis. The magnitude is indicated by the color scale on the right. Thus, each image provides a compact representation of the interruptions, freezes, amplitude decrements, and slowing that reflects the function of persons with PD [25].

## Conclusion

A low-cost method for trained raters around the world to blindly rate the signals of quantitative continuous measurements of movements of the extremities [4,5] and their transforms was presented in this article. Validation of the method with the ratings of 35 trained raters of assessments of participants with PD, MSA, and TD [8,9] was described.

A protocol has been developed to apply visual observation by trained raters to generate quantitative measurements of the accelerometry signals of movements by participants analogous to the live assessments of participants in person [[4], [5]]. The proposed protocol has been constructed to provide the means for trained raters to score the signals of movements and their transforms without seeing the movements performed by participants and without knowing the diagnosis of the participant and the laterality of movement. The protocol has been remotely conducted by 35 trained raters located around the world to produce a valuable dataset [8,9] for analysis and interpretation.

This inexpensive procedure to quantitatively measure motions in PD and other movement disorders will be a valuable resource for colleagues, particularly those in underdeveloped regions with limited budgets. The dataset will serve as a template for other investigations to develop novel techniques to facilitate the diagnosis, monitoring, and treatment of PD, other movement disorders, and other nervous and mental conditions. The procedure will provide the basis to obtain objective quantitative measurements of participants in clinical trials [10,11].

This procedure provides the basis to stratify people with PD and related conditions by patient diagnosis, disease severity, medication status, and other pertinent variables and to analyze the output of sophisticated signal processing systems to identify the pathognomonic patterns that uniquely identify different movement disorders.

This protocol provides the means for scientists, providers, patients, families, and administrators to readily assess the current function of people with PD and other movement disorders. The proposed protocol generates a foundation for experts at tertiary care centers to remotely assess the current function of patients in remote locations. Thus, providers in distant and underserved locations can be given objective measurements of the current state of their patients in order to apply the principles of precision medicine to develop optimal treatment plans for their patients with PD and related disorders.

**Glossary of nomenclature and symbols**

**Continuous wavelet transform (CWT)**: A technique to generate a compact summary of the base and harmonic frequencies and amplitudes of signals

**Deep brain stimulation (DBS):** Activation of specific regions of the brain by electrodes that have been surgically placed in precise locations

**Electromyogram (EMG**): Signals generated by muscles

**Fast Fourier transform (FFT)**: A technique utilizing sines and cosine to generate a compact summary of the frequency and amplitude of signals

**Hertz (Hz**): Cycles per second

**Inertial measurement unit (IMU)**: Instrumentation to measure inertia

**Low-cost quantitative continuous measurement of movements in the extremities of people with Parkinson's disease**[5]: An inexpensive novel structured assessment of movements of people with Parkinson's disease and related conditions for clinical and research purposes

**Movement Disorders Society-sponsored revision of the Unified Parkinson's Disease Rating Scale (MDS-UPDRS)**[4]: The gold-standard structured assessment of Parkinson's disease and related conditions for clinical and research purposes

**Multiple system atrophy (MSA)**: A neurodegenerative disorder with some symptoms and some signs of Parkinson's disease characterized by a natural history, a prognosis, and treatments different from Parkinson's disease

**Parkinson's disease (PD**): A neurodegenerative disorder afflicting 1% of individuals aged 60 and older around the world

**Second (sec)**: One sixtieth (1/60) of a minute

**Typical development (TD)**; A representation of the physical and mental growth of healthy individuals

**World Medical Association (WMA)**: An international organization to address the health of people around the the world

## CRediT authorship contribution statement

**Manuel Enrique Hernandez:** Conceptualization, Methodology, Software, Validation, Formal analysis, Investigation, Resources, Data curation, Writing – review & editing, Visualization, Supervision, Project administration. **Liran Ziegelman:** Conceptualization, Methodology, Software, Validation, Formal analysis, Investigation, Resources, Data curation, Writing – review & editing, Visualization, Supervision, Project administration. **Tanvi Kosuri:** Methodology, Validation, Formal analysis, Investigation, Resources, Data curation, Writing – review & editing. **Husain Hakim:** Methodology, Validation, Formal analysis, Investigation, Resources, Data curation, Writing – review & editing. **Luqi Zhao:** Investigation, Resources, Data curation, Writing – review & editing. **Kelly Alexander Mills:** Resources, Data curation, Writing – review & editing, Visualization, Supervision, Project administration. **James Robert Brašić:** Conceptualization, Methodology, Software, Validation, Formal analysis, Investigation, Resources, Data curation, Writing – original draft, Writing – review & editing, Visualization, Supervision, Project administration.

## Declaration of Competing Interest

The authors declare that they have no known competing financial interests or personal relationships that could have appeared to influence the work reported in this paper.
